# The mean circulatory filling pressure—physiological rationale and methodological considerations

**DOI:** 10.14814/phy2.70825

**Published:** 2026-03-16

**Authors:** Per Werner Möller, Anders Åneman, Søren Søndergaard

**Affiliations:** ^1^ Department of Anaesthesia, SV Hospital Group Institute of Clinical Sciences at the Sahlgrenska Academy, University of Gothenburg Gothenburg Sweden; ^2^ Intensive Care Unit, Liverpool Hospital, South Western Sydney Local Health District and South Western Sydney Clinical School University of New South Wales Sydney New South Wales Australia; ^3^ The Ingham Institute for Applied Medical Research Sydney New South Wales Australia; ^4^ Faculty of Health Sciences Macquarie University Sydney New South Wales Australia; ^5^ Centre of Elective Surgery Regional Hospital of Silkeborg & Institute of Clinical Medicine, University of Aarhus Aarhus C Denmark

van Loon et al. recently raised concerns regarding the experimental determination and clinical utility of the mean circulatory filling pressure (MCFP) (van Loon et al., 2025). From Weber via Starling to Guyton the definition is now given as “the pressure that would be measured at all points in the entire circulatory system if the heart were stopped suddenly and the blood were redistributed instantaneously in such a manner that all pressures were equal.” (Guyton et al., 1954; Starling, 1897; Weber, 1850). Guyton reiterated Starling's experiments, noting the need for immediate arteriovenous shunting after heart fibrillation and for determining MCFP within seconds before sympathetic activation altered vascular resistance and capacitance. The implicit purpose of the MCFP determination is to probe the prevailing volume state, or more specifically, the pressure manifestation of the stressed volume, that was valid immediately prior to the maneuver that led to pressure equilibrating to zero flow. In their study, van Loon and colleagues collected hemodynamic perimortem data (cardiac output, central venous pressure, and mean arterial pressure; −20 to +600 s) in an experimental porcine model with circulatory arrest induced in euvolemia [pentobarbital overdose, abolishing autonomic reflexes, or ventricular fibrillation (VF)], preserving autonomic reflexes, and hypovolemia (VF). These were compared with hemodynamic data from computer simulations of the dynamic responses to a sudden loss of pump function with intact or absent cardiovascular reflexes. In a dataset spanning 10 full minutes post arrest, agreement between experimental and simulated hemodynamic data improved when cardiovascular reflexes were included in the model. This allowed the authors to confirm that their experimental data did indeed reflect the reactivity of an organism exposed to circulatory disaster, yet this was inherent to the experimental design. In the light of previous research, it is not surprising that a setup without an arteriovenous shunt, a markedly late time point for crossing (30 s post arrest), and incomplete pressure equilibration would result, at best, in suboptimal MCFP estimations. We struggle to follow the authors' reasoning when they conclude that persisting arteriovenous pressure differences and blood pressure fluctuations from this point and onwards for 10 min *postmortem* could somehow invalidate the concept of stressed volume and MCFP in the running circulation. Whatever pressure you may measure in that context, it must not be mistaken for MCFP.

On the contrary, we have validated Guyton's venous return model using a series of well‐controlled physiological porcine studies. Using experimental models with intact circulation and right atrial balloon occlusion (Berger et al., 2016), veno‐arterial (VA) ECMO and ligated pulmonary artery (Moller et al., 2017), or VA ECMO with VF‐induced shock (Moller et al., 2018), we have shown that MCFP and the related mean systemic filling pressure accurately describe both absolute values and changes in stressed volume. It is important to note that several of the studies referenced in the van Loon report used the mean systemic filling pressure analogue (P_msa_) developed by Parkin and Leaning (Parkin & Leaning, 2008). The calculation of P_msa_ relies on a simpler model than the CVSim used by the authors. The P_msa_ equation can be easily adapted into a perioperative spreadsheet, enabling the calculation of the three determinants of circulation: P_msa_, representing the volume state; E_h_, the global heart efficiency [(P_msa_‐RAP)/P_msa_], representing the inotropic state (RAP: right atrial pressure); and SVR, the resistive state. Furthermore, the pressure gradient for venous return, VRdP, emerges as (P_msa_‐RAP). Sondergaard illustrated the analytic options in primary determinants and derived variables (Sondergaard et al., 2019). We have thoroughly tested the validity of P_msa_ and showed that it accurately estimated absolute and changing values of MCFP, with excellent within‐method precision (Werner‐Moller et al., 2022). The reasons put forward by van Loon et al. to challenge the practical application of MCFP seem more related to their experimental model than to the physiological construct. When determined correctly, that is, before reflex activation has changed the object of interest, MCFP is a validated pressure representation of the stressed vascular volume with the implicit intention to be applied to *the running circulation*‐quite literally a pressure concept that keeps up with the flow.

## AUTHOR CONTRIBUTIONS

Søren Søndergaard wrote the first draft. All authors revised and approved the manuscript.

## FUNDING INFORMATION

The authors have nothing to report.

## CONFLICT OF INTEREST STATEMENT

The authors declare no conflicts of interest.
